# Emotional and Sexual Adaptation to Colon Cancer: Perceptual Congruence of Dyadic Coping Among Couples

**DOI:** 10.3389/fpsyg.2022.802603

**Published:** 2022-05-09

**Authors:** Alexandra Stulz, Nicolas Favez, Cécile Flahault

**Affiliations:** ^1^Department of Oncology, Hôpital Saint-Joseph, Paris, France; ^2^Department of Laboratoire de Psychopathologie et Processus de Santé, Université Paris Cité, Paris, France; ^3^Department of Unité de Psychologie Clinique des Relations Interpersonnelles, Université de Genève, Geneva, Switzerland

**Keywords:** colon cancer, couple, congruence, dyadic coping, emotional and sexual satisfaction

## Abstract

**Context:**

Colon cancer is the 3rd most common cancer in the world. The diagnosis leads the patient and his relatives into a process of mourning for their health and previous life. The literature highlights the impact of the disease on couples. Cancer can either alter or strengthen the relationship. The disease will directly or indirectly affect both partners. Such impact starts with the diagnosis and lasts long after treatments. No study has analyzed both emotional and sexual interactions between partners throughout the illness so far.

**Objective:**

This research aims to identify and describe whether congruence within couples tends to improve emotional and sexual adjustment.

**Method:**

Thirteen couples took part in this research by answering a set of questionnaires investigating, in particular, dyadic coping strategies, marital and sexual satisfaction. Non-parametric analyses were performed on the quantitative data.

**Results:**

Emotional satisfaction is good among the couples in our study. There are important similarities in partners’ emotional adjustment. Patients who are most satisfied with their couple typically have a partner who is also satisfied. This was an expected result based on the literature. Overall, sexual satisfaction is described as average, which is either related to a low frequency of sexual intercourse, or a gap between the ideal and actual frequency of intercourse. In terms of dyadic coping, similarities within couples tend to improve emotional and sexual adjustment. Couples in which communication about stress between the patient and their partner is congruent tend to report good marital satisfaction. We found the same results for delegated coping of both the patient and the partner, and for negative coping of the partner. Sexual adjustment is linked to a similar perception within the couple of a common dyadic coping.

**Conclusion:**

Emotional and sexual adjustment is largely linked to the quality of the partner’s support. The congruence of couple support strategies has been identified as an important factor in emotional satisfaction. In addition, the more couples implement joint stress management, the better their sexual satisfaction.

## Introduction

### Context

Institutions focusing on cancer epidemiology report a particularly high incidence of colon cancer (INCa, 2017). The most recent data show that colorectal cancer was the 3rd (respectively 2nd) most diagnosed cancer in men (respectively women), in 2016.

#### Physical and Psychological Impacts

Colon cancer as well as its treatments can have significant physical repercussions: abdominal pain; transit disorder; anemia; rectorrhagia; fatigue; weight loss, loss of appetite, nausea, and vomiting; bowel obstruction or perforation (American Cancer Society, 2013).

In addition, the experience of cancer is subjective and characterized by psychological upheaval (Baillet and Pelicier, 1998; Reich et al., 2008; Dolléans, 2010). The diagnosis of cancer is often associated with intense emotional shock, incomprehension or a feeling of injustice (Baillet and Pelicier, 1998; Wisard, 2008). Cancer conveys an image of death, of emaciated bodies, of suffering (Reich et al., 2008; Altmeyer and Pinault, 2009); patients talk about their fear of dying, fear of suffering and physical mutilation. They also mention their worries about losing their place in their personal and intimate life, and their place in society (Wisard, 2008; Dolléans, 2010).

More specifically, in the case of colon cancer, the symbolism of the affected area has psychological repercussions on patients (Dolléans, 2010). Indeed, colon cancer refers to the notion of stool, fecal matter, which is taboo in our society (Altmeyer and Pinault, 2009). Patients may feel a sense of shame associated with the location of their disease and the need to discuss bowel movements in detail with healthcare teams. There are also concerns related to cleanliness, loss of sphincter control, or a fear of disgust that this might produce in others. Furthermore, treatments can be experienced as an intrusion into the body, a brutal and violent aggression (Altmeyer and Pinault, 2009). The body is most often damaged by the treatments, both in the short and long term, which can lead to psychological distress (Reich et al., 2008), a feeling of depersonalization, a loss of control over oneself and one’s identity, isolation, shame, and anger (Altmeyer and Pinault, 2009).

The impact of both the disease and the treatment reaches beyond the individual patient. The onset of cancer within a family causes both emotional and functional upheavals (Carlson et al., 2000; Sevaux, 2006; Razavi et al., 2008; Revenson and DeLongis, 2011). Spouses are often the primary “caregivers,” very commonly cited as the most important source of support by patients (Baider et al., 1996; Pitceathly and Maguire, 2003; Stuhlfauth et al., 2018). The relationship between a patient and their partner is complex and differs from the supportive relationships held with family and friends. Partners have a supportive role in the healing process, sharing in the daily routine (Lory, 2010).

Spouses may experience significant existential stress knowing their partner is ill. The diagnosis of cancer, let alone the mere use of this term, awakens anxieties about death, uncertainty, suffering, loss, and separation (Reynaert et al., 2006; Proia-Lelouey and Lemoignie, 2012). As a key source of support for patients, they are also the most affected by the disease (Razavi et al., 2008). The literature highlights the frequent presence of psychological distress and anxiety in partners, sexual dysfunction and/or decreased libido, loss of mobility and decreased leisure activities, or difficulties in marital communication and romantic relationships (Libert et al., 2006; Braun et al., 2007).

#### Cancer and the Interdependence of Partners: «We-Disease»

The psychological and relational adjustment of couples is increasingly studied in the field of cancer. Research focuses on patient and partner distress, support, cohesion, relational satisfaction, and communication within couples (Acitelli and Badr, 2005; Kayser et al., 2018), most often in the context of breast or prostate cancer (Proia-Lelouey and Lemoignie, 2012). However, the majority of studies still focuses on patients and partners independently, omitting the relational consequences for the couple as a system (Acitelli and Badr, 2005). Here we wish to focus on the dyadic dimension and congruence within couples in adjusting to illness.

Interdependence can be defined as the mutual influence of two partners, acting as a unit rather than as two independent individuals (Kayser and Scott, 2008). The couple as an interpersonal and interdependent system can contribute to individual wellbeing, improve the effectiveness of adjustment, create a safe environment where partners can talk to each other, express their needs, recognize each other’s strengths, change the meaning of the illness and allow for effective coping strategies to be implemented. Conversely, the relationship can participate in the development or maintenance of psychological suffering when difficulties arise and persist, both at the individual or couple level (Bodenmann, 2005; Kayser et al., 2007; Kayser and Scott, 2008; Drabe et al., 2015; Feeney and Collins, 2015).

When faced with illness, two distinct patterns of reaction by couples have been described (Acitelli and Badr, 2005; Kayser et al., 2007; Kayser and Scott, 2008):

- “mutual reactivity”: the illness is experienced as a common stressor, affecting both partners. This type of pattern allows for individual and couple developments, strengthens the relationship, increases intimacy;
- “avoidance–indifference”: the illness is perceived as an individual stress, referring only to what is experienced by each partner separately, not taking into account the experience of the other. This type of pattern brings little evolution to the couple, it can, however, bring personal evolution.

In general, couples who perceive illness as a “couple experience” report better individual and dyadic adjustment to illness and greater relationship satisfaction than those who perceive illness as an individual stressor (Acitelli and Badr, 2005; Ahmad et al., 2017).

#### Congruence Within Couples

Partner interdependence raises the question of congruence in partner responses to adjustment to cancer. This field of research was developed in the 1980s and focuses on the effects of congruence on the emotional adjustment of patients and partners, mostly in breast cancer (Kraemer et al., 2011; Meier and Cho, 2019). A few studies have subsequently applied the concept of congruence to marital satisfaction (Badr, 2004; Norton and Manne, 2007; Meier and Cho, 2019). These works assess how similarities in the perception and/or coping mechanisms put in place by partners affect their individual and relational adjustment (Iafrate et al., 2012; Falconier and Kuhn, 2019).

Congruence can be assessed by looking at the degree to which partners agree on how they cope as a couple or “perceptual congruence of dyadic coping.” It is an assessment of the recognition of each partner’s efforts, measured at the couple level (Meier and Cho, 2019).

A look at the current literature tells us that the congruence effect between partners depends on the variables studied (Falconier and Kuhn, 2019). For example, couples reporting high congruence in their “perceived dyadic adjustment” showed greater marital satisfaction with no effect on individual distress (Iafrate et al., 2012). Badr (2004) adds that congruence can be helpful depending on the type of coping strategy implemented: couples who are more congruent in active engagement and more complementary in protective and avoidance behaviors report greater marital satisfaction.

Results in the field of cancer are heterogeneous and have never been applied to colorectal cancer. Moreover, the evaluation of congruence only concerns individual adjustment or marital satisfaction, without looking at sexual adjustment. Our study thus proposes to pay attention to the congruence in the emotional and sexual adjustment of couples where one of the members has colon cancer.

### Objectives

This research aims to identify and describe congruence within couples that may impact emotional and sexual adjustment.

In order to meet our objective, we defined specific research questions touching upon the following aspects:

- RQ1: Marital and sexual satisfaction of couples in our sample. This research question is exploratory; we wish to observe partner satisfactions. However, we expect to observe rather high levels of satisfaction within the couples, which we will discuss more specifically in the Section “Discussion.”
- RQ2: Intra-couple correlations of marital and sexual satisfaction to observe congruence between partners. We expect partners to exhibit a high degree of similarity in marital and sexual satisfaction.
- RQ3: Correlation between congruence of dyadic coping strategies and marital and sexual satisfaction. We expect a positive correlation between congruence and satisfaction: the more partners report congruence, the more satisfied they are with their marital and sexual life.

## Materials and Methods

### Methods

This research is a quantitative, cross-sectional study of couples in which one of the partners has colon cancer.

The recruitment of patients and their partners took place in the oncology department of the Groupe Hospitalier Paris Saint Joseph. Participation in this study was open to all patients (men and women) diagnosed with colon cancer, during chemotherapy or in remission.

Patients meeting all study inclusion criteria (Table 1) were identified within the oncology or digestive surgery department. Identification was performed routinely between March 1, 2019 and March 31, 2020. Patients were then offered participation in the research *via* physical mail sent to their home and telephone contact. After oral acceptance of participation, all the necessary documents for the study were sent to the patients (information documents, consent, and questionnaires). Through them, the study was proposed to their partner.

**TABLE 1:** Inclusion criteria.

	Patients	

*Inclusion criteria*	*Non-inclusion criteria*
Patient with colon cancer	Being unable to answer the questions (e.g., cognitive impairment)
Patient who has been in a relationship for more than 2 years	Presence of a psychiatric disorder modifying the relationship to reality or impairing participation in the study
Patient over 18 years old and able to read French	
Patient who has given free and informed consent	

	**Partners**	

***Inclusion criteria***	***Non-inclusion criteria***

Partner over 18 years of age and able to read French	Cancer in treatment or in remission
Partner has given free and informed consent	Inability to answer questions (e.g., cognitive impairment)
	Presence of a psychiatric disorder that modifies the relationship to reality or hinders participation in the study.

### Materials

#### Sociodemographic and Medical Questionnaire

We created a socio-demographic questionnaire including information on gender, role (patient or partner), age, marital status, number of children, socio-professional status. Information about psychological and/or sexological monitoring was also collected. We also obtained relevant medical information for this study from the patients’ medical records (treatments, dates).

#### Dyadic Coping Questionnaire

Bodenmann (2008) Dyadic Coping Inventory (DCI) assesses dyadic coping within couples. This tool measures the degree of support and help provided and perceived by each spouse during a stressful event. Specifically, it assesses communication around stress, positive dyadic coping (including supportive, delegative, and shared coping), negative dyadic coping, and perceived effectiveness of couple stress management. This scale also includes an assessment of marital satisfaction. The scale has been used in many previous studies, it has good psychometric qualities in its French version (α between 0.64 and 0.89–only the negative dyadic coping subscales have lower α at 0.50 and 0.53, which we take into account in our analysis) (Ledermann et al., 2010). In our sample, α’s ranged from 0.75 to 0.93, with the negative dyadic coping scales exhibiting higher values than expected (0.62 and 0.76, respectively, for patients and their partners).

#### Sexual Functioning Questionnaire

We chose the Derogatis Sexual Functioning Inventory (DSFI) by Derogatis and Melisaratos (1979), translated into French by Gauthier and Garceau (1982). This questionnaire is divided into 10 subscales: Information, Recent Experiences, Drive, Attitudes, Psychological Symptoms, Emotions, Role Definition, Fantasy, Body Image, and Sexual Satisfaction (overall score). The subscales can be used separately. For our study, we used only the Recent Experiences, Sexual Satisfaction, and Global Sexual Satisfaction Index scales. The tool has good psychometric qualities in its original version with α’s ranging from 0.60 to 0.97 (Géonet et al., 2017). In our study, the α’s range from 0.60 to 0.78.

### Statistical Analysis

Non-parametric analyses were performed on the quantitative data. In order to respond to RQ1, descriptive analysis (mean, standard deviation) was conducted on marital and sexual satisfaction of couples; in order to respond to RQ2, Spearman correlations were performed between each partner’s marital and sexual satisfaction within the same couple. Finally, in order to respond to RQ3, we first created a congruence score for each couple (delta between the patient’s and partner’s score). This score was then correlated with marital and sexual satisfaction.

### Ethical and Deontological Aspects

The study involving human participants was reviewed and approved by the Conseil d’évaluation éthique pour les recherches en santé (CERES) on September 13, 2016. The delay between the committee’s approval and the actual start of our research (March 2020) was linked to the organization of doctoral work and the setting up of hospital partnerships.

We informed the participants of the anonymity of the data provided, as well as of their right to refuse or withdraw from the study, without any consequence on their medical monitoring.

## Results

### Descriptive Data

All 13 couples in our study were 56 years old on average (min: 33, max: 80, sd: 13.89). They had been in a relationship for 27 years (min: 4, max: 47, sd: 14.92) and had on average 2.75 children per couple. Ten couples were married or in a civil union, while 3 were cohabiting. Finally, the vast majority of couples lived together, with only one couple living separately (the patient’s spouse was working abroad) (Table 2).

**TABLE 2:** Descriptive data.

	Full sample		Patients		Partners		*p*
	(*N* = 26)		(*N* = 13)		(*N* = 13)		
**Age** (mean [*sd*])	56,50 [*13,89*]		56,08 [*13,91*]		56,92 [*14,41*]		*ns*

	***N***	**%**	***N***	**%**	***N***	**%**	

**Couple Status**							/
Cohabitation	6	8,0	/	/			
Married/Partnered	20	92,0					
Duration (mean)	26,62						

**Children**							/
Yes	17	65,4	/	/			
Number	2,75						

**Level of study**							*ns*
No diploma	1	3,8	1		0		
BEPC, CAP, BEP, or equivalent	6	23,1	3		3		
High school diploma or équivalent	1	3,8	0		1		
High school diploma +2	3	11,5	1		2		
Bachelor’s degree and more	15	57,7	8		7		

**Professional status**							0,039
In activity	11	42,3	3		8		
Leave of absence from work	5	19,2	5		0		
Looking for a job/not working	1	3,8	0		1		
Retired	9	34,6	5		4		

A comparison between patients and partners indicates that they differ only in professional status, partners being significantly more active than patients.

Finally, all the patients were diagnosed at stage 3 (62%) or 4 (38%), and about half of our sample was undergoing treatment (46.2%). All of them received chemotherapy, 12 of them also underwent surgery and only one received radiotherapy (Table 3).

**TABLE 3:** Medical data.

	Patients (*N* = 13)	
	*N*	%
**Cancer stage (TNM)**		
3	8	61,5
4	5	38,5
**Treatment status**		
In process of chemotherapy	6	46,2
In remission	7	53,8
**Treatments**		
Surgery	12	92,3
Chemotherapy	13	100
Radiotherapy	1	7,7
Stoma	1	7,7
**Time since diagnosis**	1,977 year [*sd: 1,51*]	

#### RQ1: Descriptive Analysis of Marital and Sexual Satisfaction of Couples

Couples described themselves as quite satisfied with their relationship (*M* = 4, sd = 1.2, on a Lickert scale of up to 5). Just over three-quarters of the participants reported being very or extremely happy. About 10% reported being somewhat or extremely unhappy.

Since the disease, half of the participants report having sex less than once a month, with almost 20% never having sex. It is interesting to note that the actual frequency of intercourse is significantly correlated with sexual satisfaction (*r* = 0.588, *p* < 0.05): the less frequent the intercourse, the less satisfied they are. The degree of difference between actual and desired frequency of intercourse is strongly correlated with sexual satisfaction (*r* = −0.519, *p* < 0.005): the greater the difference between actual and desired, the less satisfied the partners are with their sexuality (Table 4).

**TABLE 4:** Correlation between the variables of the DSFI (couples).

		1	2	3	4
1. Actual frequency of sexual intercourse	Correlation coefficient	1,000	,422*	−,456*	,588**
	Sig. (2-tailed)	.	,032	,019	,002
2. Ideal frequency of intercourse	Correlation coefficient		1,000	,596**	−,002
	Sig. (2-tailed)		.	,001	,992
3. Differences between actual and ideal frequency	Correlation coefficient			1,000	−,519**
	Sig. (2-tailed)			.	,007
4. Sexual satisfaction	Correlation coefficient				1,000
	Sig. (2-tailed)				.

**Correlation is significant at the 0.01 level (2-tailed).

*Correlation is significant at the 0.05 level (2-tailed).

### Perceptive Congruence Between Partners: Analysis of the Correlation Between Partner Data

#### RQ2: Congruence of Marital and Sexual Satisfaction

There was a strong positive and significant correlation between the marital satisfaction scores of the patients and their partners (*r* = 0.617, *p* < 0.05): the more positively the patients evaluated their marital relationship, the more positively their partner evaluated it. There was also a positive and significant correlation between the sexual satisfaction scores of patients and their partners (*r* = 0.638, *p* < 0.05): the more patients evaluated their sexuality in a positive way, the better the evaluation by partners of their own sexuality (Table 5). When there are differences between partners, patients always report slightly more satisfaction with their sexuality than spouses.

**TABLE 5:** Patient-partner correlation of marital satisfaction/sexual satisfaction.

	Patients	
Partner	Marital satisfaction	Sexual satisfaction
Marital satisfaction	,617*	/
Sig. (2-tailed)	,025	
Sexual satisfaction	/	,638*
Sig. (2-tailed)		,019

*Correlation is significant at the 0.05 level (2-tailed).

#### RQ3: Dyadic Coping and Marital and Sexual Satisfaction

Dyadic coping variables require preliminary work to facilitate the analysis of similarities.

- Communication around stress, supportive, negative, and delegated dyadic coping subscales:
  • Patients and partners rated both their own and their spouse’s coping
  • We chose to create a variable assessing the similarity of perception by partners (e.g., the difference between patient and spouse scores on patient communication)
- Common dyadic coping subscales, coping effectiveness assessment, and total dyadic coping:
  • Patients and partners rated only their own perception
  • We created a simple variable to measure the difference between patient and partner scores.

The degree of similarity between what one partner does and their spouse’s perception of it (e.g., how I communicate and what my partner perceives of my communication) is related to marital satisfaction for several of the dyadic coping subscales. Indeed, there are negative and significant correlations with patient (*r* = −0.452, *p* < 0.05) and partner (*r* = −0.576, *p* < 0.01) communication perceptions, partner negative coping perceptions (*r* = −0.582, *p* < 0.01), and patient (*r* = −0.413, *p* < 0.05) and partner (*r* = −0.473, *p* < 0.05) delegated coping perceptions. Two trends were identified for all other perceptions (cf. Table 6).

**TABLE 6:** Correlation between delta of dyadic coping strategies and adjustment variables.

		Marital satisfaction	Sexual satisfaction
Delta perception of patient communication	Correlation coefficient	−,452*	−,220
	Sig. (2-tailed)	,048	,280
Delta perception of partner communication	Correlation coefficient	−,576**	,027
	Sig. (2-tailed)	,002	,895
Delta perception of patient’s supportive coping	Correlation coefficient	−,377	−,339
	Sig. (2-tailed)	,058	,090
Delta perception of partner’s supportive coping	Correlation coefficient	−,341	−,160
	Sig. (2-tailed)	,088	,436
Delta perception of patient’s negative coping	Correlation coefficient	−,387	−,202
	Sig. (2-tailed)	,051	,323
Delta perception of partner’s negative coping	Correlation coefficient	−,582**	−,044
	Sig. (2-tailed)	,002	,832
Delta perception of patient’s delegated coping	Correlation coefficient	−,413*	−,278
	Sig. (2-tailed)	,036	,170
Delta perception of partner’s delegated coping	Correlation coefficient	−,473*	−,267
	Sig. (2-tailed)	,015	,188
Delta common dyadic coping	Correlation coefficient	−,220	−,417*
	Sig. (2-tailed)	,280	,034
Delta dyadic coping assessment	Correlation coefficient	−,321	−,170
	Sig. (2-tailed)	,109	,406
Delta total perceived dyadic coping	Correlation coefficient	−,173	−,090
	Sig. (2-tailed)	,399	,663

**Correlation is significant at the 0.01 level (2-tailed).

*Correlation is significant at the 0.05 level (2-tailed).

Finally, sexual satisfaction was only negatively and significantly correlated with the gap in joint dyadic coping assessed by partners (*r* = −0.417, *p* < 0.05). This means that the more the two partners of the same couple differ in their implementation of common strategies, the less satisfied they are with their sexuality. No other correlation was significant (Table 6).

## Discussion

Colon cancer is one of the most frequently diagnosed cancers in the world, for which screening and treatment currently allow a longer survival. However, all of the treatments available today lead to a significant alteration in the quality of life of both patients and partners. The couple will experience the onset of the disease, often modifying its functioning. These global repercussions will persist for several years. We felt it was necessary to look at the emotional and sexual experiences of these couples affected by colon cancer.

### Couples (RQ1)

In our sample, couples described themselves as happy or very happy in their relationship. We expected couples to describe themselves as happy, as studies of couples often have a selection bias: happy couples are more likely to participate. However, sexual satisfaction is described as average in couples, due to the low frequency of sexual intercourse and a significant gap with the ideal frequency of intercourse. This alteration in sexual satisfaction is a common consequence of cancer and related treatments, and is recognized as one of the most persistent impacts after treatment (Traa et al., 2012).

### Congruence

Congruence in the emotional and sexual adjustment of couples in which one member has colon cancer has never been evaluated in the literature.

#### Emotional Adjustment and Marital Satisfaction (RQ2)

We have seen that there are important similarities between partners within couples. Thus, the partners of patients who are most satisfied with their relationship also tend to be satisfied themselves. This result was expected, due to the couple-focused theme of our research and the enrollment of partners through patients. Two couples still rated their marital satisfaction very negatively, with a strong similarity as well.

In other words:

*“When I am satisfied with our relationship as a couple, you are too. If I’m not, neither are you.”*

#### Sexual Adjustment (RQ2)

Although there is a correlation between the sexual satisfaction of patients and partners, highlighting similarities between the partners, differences still exist. When there are differences between partners, patients always declare themselves slightly more satisfied with their sexuality than spouses. It can probably be explained by different expectations between patients and partners on sexuality (Almont et al., 2019): patients tend to put sexuality on hold at the time of diagnosis and during treatment, in relation with the shock of the diagnosis, the death anxiety, and the implementation of treatments altering quality of life. Putting sexual issues on hold seems easier for patients–who are focused on treatment–than for their partners.

In other words:

*“When I am satisfied with my sexuality, you tend to be too”*

#### Dyadic Coping (RQ3)

Similarities within couples in dyadic coping tend to facilitate marital and sexual adjustments.

Thus, when patient and partner communication is well perceived by both members of the couple, they are more satisfied with their marital relationship. More broadly, our results emphasize the amount and need of communication within couples (Badr, 2004; Barnoy et al., 2006). A couple in which one partner reports a great deal of illness-related communication while the other does not perceive it will tend to do worse from a relationship standpoint, due to the latter’s difficulty to adjust to the needs of their partner.

On the other hand, when patients and partners perceive the partner’s negative coping strategies in the same way, then marital satisfaction is better. These results are interesting, as negative coping strategies are usually associated with lower marital satisfaction in the literature. This would mean that the similarity of perception in couples regarding these strategies actually promotes better marital adjustment. Congruence could thus provide benefits, even when identified on negative strategies (Bodenmann et al., 2011).

Finally, the correct perception of delegated coping strategies by patients and partners is related to the good marital satisfaction of both partners. In other words, if the patient asks for help and perceives that his or her partner is providing it, marital satisfaction is better. Similarly, if the partner is supportive and the partner perceives that he or she is supportive, then marital satisfaction is higher.

Congruence of support strategies in the couple has been identified as an important factor in marital satisfaction in several studies of different types of cancer, including colorectal cancer (Barnoy et al., 2006; Norton and Manne, 2007; Meier and Cho, 2019). For women followed for breast cancer (Ben-Zur et al., 2001), it is primarily in the area of supportive coping (including emotional support) that congruence plays a positive role on affective adjustment in patients. Bodenmann et al. (2011) also emphasize that congruence in supportive coping is related to marital satisfaction; they, however, mention that congruence between partners is less predictive of marital satisfaction than initial coping strategies. In our sample of couples, congruence in supportive coping strategies was not found, suggesting that the effect of supportive behavior is more efficient than the perception of congruence within couples in couples’ emotional adjustment.

Taken together, these correlations tell us that similarities in partners’ perceptions of dyadic communication and coping strategies promote marital satisfaction. In other words, “the more my partner and I perceive the same thing about each other’s behavior, the more likely we are to be satisfied with our relationship.”

Lastly, sexual adjustment is linked to a similar perception of a common dyadic coping within the couple. Common dyadic coping strategies are all the behaviors and cognitions of the couple, in order to manage the stress of the illness. These strategies emphasize the commitment of both partners to the relationship. Thus, the more couples implement joint stress management, the more sexually satisfied they are.

### Limits

There are limitations to this work that we would like to discuss. First of all, the inclusion of patients as soon as they are diagnosed with cancer remains laborious, be it for medical clinical trials or psychosocial studies. The shock of diagnosis, death anxiety, pre-treatment anxiety and uncertainty about the future probably limit these inclusions. Moreover, recruitment of couples is not easy. Dyadic studies often face a low participation rate of couples who, when they do participate, generally report very good marital satisfaction (Manne et al., 2004; Segrestan-Crouzet, 2010; Hagedoorn et al., 2011).

In addition, there is a selection effect here that should be noted, as partners are recruited through patients. We are aware that this leads to the recruitment of couples who are mostly satisfied with their relationship. Nonetheless, it remains clinically relevant to try and identify factors that favor a good adjustment of couples to the disease.

Finally, the topic of sexuality is an additional barrier, being a taboo in itself. Indeed, talking about sexuality remains difficult, for both patients and caregivers (Annerstedt and Glasdam, 2019; Traumer et al., 2019). The number of couples included in our research is therefore limited. This makes it difficult to generalize our results to all colon cancer patients and their partners. This also reduces the statistical power, limits the choice of our analyses and therefore requires attention in the interpretation of the results.

In addition, the questionnaires used in our study do not include questions about life events that patients and partners may be going through outside the disease. As our study recruited patients several years after diagnosis, it is possible that other life events may have influenced our results.

## Conclusion

The originality of this study was to focus on couples and to analyze their emotional and sexual adjustment during and after treatment.

Stress management within couples is an important variable in the adjustment of patients and partners (Untas et al., 2009). First, communication between partners regarding individual needs and attention to each other is quite high and frequent. It has positive effects especially when it is congruent and corresponds to each other’s needs. The majority of couples use positive behaviors, favoring joint management of the disease and treatments. Negative behaviors are rarely used, and remain associated with an alteration in emotional and sexual adjustment. All of these results are also found in the literature regarding overall emotional adjustment, particularly in breast cancer (Segrestan-Crouzet, 2010; Stulz et al., 2014). In addition, we note a link between patient and partner adjustment, and strong similarities within couples on many variables. Congruence within couples tends to promote emotional and sexual adjustment, so couples tending to react in similar ways seem to do better than couples reacting in very divergent ways to the disease. These findings are clinically interesting, but remain to be confirmed, especially as the literature continues to emphasize the superiority of coping strategies *per se* over perceived congruence as a predictor of couples’ affective adjustment (Bodenmann et al., 2011; Regan et al., 2015).

## Data Availability Statement

The original contributions presented in the study are included in the article/supplementary material, further inquiries can be directed to the corresponding author.

## Ethics Statement

The study involving human participants was reviewed and approved by the Conseil d’évaluation éthique pour les recherches en santé (CERES) on September 13, 2016. Written informed consent to participate in this study was provided by both the patient and the spouse.

## Author Contributions

AS contributed to conception and design of the study and the acquisition, analysis, or interpretation of data for the work, drafted the work, organized the database, performed the statistical analysis, wrote the first draft of the manuscript, and wrote sections of the manuscript. NF and CF revised the work critically for important intellectual content. All authors contributed to manuscript revision, read, and approved the submitted version.

## Conflict of Interest

The authors declare that the research was conducted in the absence of any commercial or financial relationships that could be construed as a potential conflict of interest.

## Publisher’s Note

All claims expressed in this article are solely those of the authors and do not necessarily represent those of their affiliated organizations, or those of the publisher, the editors and the reviewers. Any product that may be evaluated in this article, or claim that may be made by its manufacturer, is not guaranteed or endorsed by the publisher.
